# Some Observations on the Possibilities of Preventive Surgery

**Published:** 1889-10

**Authors:** E. J. Ward

**Affiliations:** Waxahachie, Texas


DANIEL'S

### TEXAS MEDICAL JOURNAL.

A Representative Organ of the Medical Profession, and an Exponent of Rational Medicine; devoted to the Organization, Advancement and Elevation of the Profession in Texas.

PUBLISHED MONTHLY.—SUBSCRIPTION $2.00 A YEAR.

Vol. V. AUSTIN, OCTOBER, 1889. No. 4.

### ORIGINAL ARTICLES.

CONTRIBUTED EXCLUSIVELY TO THIS JOURNAL.

*The Articles in this Department are accepted and published with the understanding that we are not responsible for, nor do we indorse the views and opinions of the writers by so doing.*

### SOME OBSERVATIONS ON THE POSSIBILITIES OF PREVENTIVE SURGERY.

BY E. J. WARD, A. M., M. D., WAXAHACHIE, TEXAS.

[Read at the Twenty-first Annual Convention Texas State Medical Association, at San Antonio, April, 1889.]

IN ALL civilized nations the preservation of human life is justly regarded as the first and highest duty of the State, and though imperfect and inadequate as are the means employed to this end, indeed must necessarily be, in the present chaotic state of our knowledge, not only of the nature and cause of disease, but of the subtle and intricate machinery of our being still with each decade there is an appreciable advance in the development of means for the conservation of life, the preservation of health, and the prevention of disease.

In one way or other almost every department of knowledge has been made tributary to the maintenance of health and the prolongation of life; and hence, notwithstanding the many additional dangers, which menace life through the constantly increasing artificial habits of the people; the multiplication of accidents which inevitably attend the growing facilities for rapid locomotion; the sacrifice of human life by the improved engines of war, and the lengthening of the death-roll by the compounding of inherited disease, the vital statistician is enabled to give to the man of to-day the cheering intelligence that "the days of his years" are noticeably lengthening.

When Henry and Morse were engaged in their toilsome experiments in telegraphy, they little dreamed that they were opening up an avenue through which the important science of meteorology was to be developed, a science which to-day compels the storms, so destructive to human life upon land and sea, to yield up the secrets of their genesis and in some measure, at least, rob them of their life-destroying power.

The early opticians could have had no conception, no matter how prophetic may have been their pre-vision, of the revelations their optical devices were destined to make, of the stupendous influence the microscope would some day exert upon therapeutics and preventive medicine.

Nor could the men who in days past, laid the foundation of the now magnificent pile to which we give the name biology, have had the faintest glimpse of the splendid discoveries of Pasteur and Koch. Doubtless they were as far from foreseeing these important achievements, as we of to-day are from estimating the practical results that may flow from them in the future, results it may be, that will open the way for the complete suppression of many or most of the diseases which now swell our mortuary reports. Already preventive medicine has done much to retard the march of disease, and to diminish the fatality of many of those which it fails to prevent.

All sciences must pass through certain stages before any useful application of them can be made. First, a great many facts must be collected, and these must be arranged before the stage of induction is reached, while the highest attainable stage, the deductive, is a still later achievement. In preventive medicine the collection and arrangement of facts necessary to serve as a basis for induction, is attended with the rarest difficulties, requiring a vast amount of technical knowledge, covering almost the whole field of scientific research. Hence, progress is necessarily slow.

In preventive surgery, of which it is my purpose to speak to you to-day, little or nothing has been done even in the preliminary stage. Is there nothing to do in this field? It seems to me that "the harvest truly is great, but the laborers are few."

That the various forms of cancer are alarmingly increasing; that uterine displacements now more than ever before menace the health and happiness of our women; that there is a larger percentage of sufferers from hernia now than in any preceding age of the world, and that many other surgical diseases are steadily gaining upon our race, cannot be doubted. The reasons for the increase of these affections are not far to seek.

In order to confine this paper to the limits which this Association in its wisdom has set, I must needs eliminate from the discussion several surgical diseases which it would be desirable to consider, and the few I shall be able to bring under your notice in the time allotted to me, must be treated with a brevity not consistent with that clearness which should characterize a paper presented for the consideration of so learned a body as the one I have the honor of addressing to-day.

It is essential to this discussion that I should direct your attention to some of the general facts of Organic Evolution, and later it will be necessary to consider very briefly, but with more particularity, one of the factors by which evolution of the organic world has been brought about, viz: the inheritance of functionally-produced modifications.

We find a large number of women, a vast number it must be regretfully admitted, who are suffering from some of the forms of uterine displacement. Why is this? Manifestly it is because the uterine ligaments and vagina are inadequate to sustain the organ in its proper pelvic position. But why this insufficiency of uterine support? To this question a majority of gynecologists will answer: "Artificial modes of life have impaired the strength of the ligamentous supports, which in women of normal health and whose functions are performed naturally, are ample to maintain the uterus in place."

We think the answer contains a two-fold fallacy. It is no doubt true that the artificial habits of women, especially those which tend to impairment of muscular tone, predispose to displacements, but the answer is only true to this extent. We shall maintain that the uterine supports of woman are naturally insufficient to maintain the womb, under all conditions, in its proper pelvic plane since the woman has attained the upright position. Hence, that the uterus of woman is naturally prone to displacement, and that the most perfect woman (I do not say the ideal woman) physically considered, is liable, under undue exertion or other slight cause, to malposition of the womb, and that the modifications resulting from the aberrations of function which accompany and are inseparable from these displacements, are transmitted to offspring. This we believe, affords the true explanation of the prevalence of uterine disorders in women, and at the same time suggests the means by which they may in the future be overcome. There can be no doubt that the inherited tendency to falling of the womb is in some degree offset by that other important factor of organic evolution, natural selection, or the survival of the fittest. But we have no means of estimating to what extent the latter factor may modify the inherited predisposition to uterine displacements. Among savages, especially those of nomadic habits, and those among whom woman is the bread winner, it no doubt plays a somewhat important role; but among civilized women, whose modes of life are such as to enable them to endure these ailments, aided by the skill of the gynecologist, its influence must be very inconsiderable indeed. The latter are often enabled to struggle through the whole or part of the child-bearing period, and thus transmit to their offspring their own unfortunate weaknesses.

Cervical lacerations, especially those which retard or prevent involution, favor, among other evil consequences, uterine displacement. This will, of course, be admitted by all gynecologists; but we shall go a step further, and say that the alterations of function, born of these displacements, result in the acquirement of modifications, which are inheritable. The evidence relied upon to support this conclusion, will be briefly discussed further on.

Let us now turn our attention for a few moments to hernia, and for the sake of convenience, we will limit our inquiries to inguinal hernia. Here, as in the case of the uterus in woman, we shall maintain that it is perfectly obvious that, in respect of his inguinal canal, man is ill fitted for the vertical position. There is certainly no proper adjustment of this part of his anatomy to the position he has assumed as the "lord of creation." The most perfect man, who to-day walks the earth, is not secure from rupture. An imperfectly closed abdominal ring constantly invites a knuckle of intestine to enter—a sort of "will you walk into my parlor, said the spider to the fly," invitation—which is, alas, too often accepted—how often, the heaps of trusses, which greet the eye in every drug store and surgical instrument shop, bear melancholy witness.

Whence all these hernias? Here again the surgeon answers, "Artificial life, predisposing to hernia," and again we reply, this is only true in part.

But the objector will say that the savage is but rarely a sufferer from hernia. This is true, and for substantially the same reasons that savage women infrequently have uterine prolapse.

If a hernia develop in the savage, he has no adequate means of keeping the gut in place, hence strangulation and death are almost inevitable, while the more favored survive to beget offspring in whom there is no hereditary predisposition to hernia, beyond the natural susceptibility already pointed out as common to all men, as at present constituted.

What surgeon has failed to recognize the traces of heredity in studying the etiology of varicocele? What anatomist has not been impressed with the anatomical incongruity of the vascular apparatus concerned in varicocele, with the erect posture of man?

The vast majority of sufferers from this affection who apply to the surgeon for relief, are persons whose vocations render necessary the upright posture, a fact which would seem to negative the theory of Gould, that varicocele is due to venous hypertrophy, resulting from the transference, at puberty, of stimuli from the testicle to the veins. If this were true, varicocele would be as likely to occur in persons of one vocation as another, and would show no preference for one side more than another; whereas the fact is, that in immensely the larger number of instances it occurs upon the left side.

For want of space I must resist the allurements to discuss the bearings of these facts upon the phylogeny of man, and content myself with merely calling your attention to the significance of the anatomical peculiarities of the left spermatic vein. When we take into consideration the length of this vessel, the angle it makes to empty its contents into the renal vein, the obstruction it meets with from the sigmoid flexure and the inadequacy of its valvular safeguards, it is certainly not difficult to see why dilatation of the spermatic plexus should occur even in the most perfect man. Nor is it a matter for surprise that varicocele, thus produced, should give rise to such modifications of function as would beget in offspring a tendency to varicocele.

Now, before discussing the question of preventive surgery, as applied to the surgical affections I have designated, it will be convenient, if not necessary, to consider, in the briefest manner possible, some of the facts upon which biologists rely as evidence that functionally-acquired modifications are inheritable.

It is well known to every one who is at all familiar with biological literature, that Lamarck and Erasmus Darwin regarded this factor as perhaps the sole agency in the evolution of the organic world, but it remained for Charles Darwin to make clear the stupendous importance of natural selection as an evolutional factor. Since then, the overshadowing importance of this factor has so obscured the one proposed by the elder Darwin, that the latter was, for a time, almost ignored; not, nowever, by Charles Darwin himself. It is probable, however, that every biologist of the present day admits the importance and necessity of this factor in organic evolution.

It would seem quite impossible to account for the small size of the jaws of civilized man, as compared with those of his remote ancestors, in any other way than by the hypothesis of the hereditary acquirement of modifications which were wrought through the partial disuse of the jaws in the eating of food so prepared as to reduce the effort of mastication to the minimum. Certainly the attenuation of the jaws in no way specially fits man to struggle against his environment; hence, we may eliminate what Mr. Herbert Spenser calls the survival of the fittest, as a factor in bringing about such attenuation, and sexual selection may, for reasons that are sufficiently obvious, be left out of consideration.

In dogs which have been bred as household pets, as the pug, the diminution in the size of the jaws and the attenuation of the muscles of mastication are so pronounced and significant as to leave no doubt that dainty feeding and consequent functual inactivity of the jaws afford the only reasonable explanation of the phenomenon. And if the changes thus wrought accumulate, generation after generation, as it is perfectly evident they do, it is certainly inferable that the inheritance of functionally-acquired modifications is the channel through which they are brought about.

The disappearance of the sesamoid bones in the hands and feet of the white man, which still exist in the negro, would apparently be inexplicable upon the hypothesis of natural or sexual selection. The disappearance of these bones, which are but vestiges of man's arboreal life in the remote past, from the hands and feet, cannot in any way enhance the chances of the white man for survival, and no reasonable explanation of their absence seems possible other than that functional inactivity of these bones, consequent upon the abandonment of arboreal habits, has resulted in their modification, and that the accumulation of these modifications through heredity has eventuated in their obliteration. The sesamoid bones here alluded to are exceptionally found in the white man, while they are uniformly present in the negro. In my own dissections I have never found them in the white man, except upon the thumbs and great toes, where they are always present, nor have they ever been absent from any of the fingers and toes of the negro.

The evolution of the race horse affords striking and seemingly irrefutable evidence of the transmission of functionally acquired modifications. The careful and persistent training of the racer tends to develop every organ and muscle necessary to rapid locomotion, and the structural modification which this functional discipline begets, is transmitted from sire and dam to offspring. The history of the running and trotting horse bears ample witness of the truth of this conclusion.

The pointer pup is without difficulty taught to point the game, and the young setter needs little or no training to teach him to set. The bull dog could not in a life time be trained to do either. The conclusion seems to be inevitable that the structural and other changes resulting from training, have, from generation to generation, been transmitted in constantly increasing increments, until the evolution of these various types was effected.

The hereditary acquirement of insanity admits of no reasonable doubt. Examination of the brains of certain insane persons shows a marked derangement of the cerebral blood vessels. In some instances it has been found that the posterior lacerated foramen was so contracted as to impede the exit of blood from the brain by way of the jugular vein, and also that pronounced pathalogical changes had taken place in the choroid plexus. These facts are full of suggestions to the biologist and clinician.

Moxon some years ago called attention to the probable influence of the choroid plexus in maintaining intra-cranial blood vascular equilibrium. These finger-like processes lying in contact with the tensely stretched pneumogastrics, become filled with blood when we lie down or stoop, and thus exert sufficient pressure upon the vagi to slow the cardiac revolutions, and in this way lessen the arterial blood supply to the brain. And as I pointed out a few years ago in a paper read before the Ellis County Medical Society, a similar influence would be exerted upon the pneumogastrics in their passage through their foramen lacerum posterius, by the accumulation of blood in the gulph of the jugular vein. It is easy to see how a pathalogical condition of these parts would affect a blood supply to the brain and bring about disturbance of cerebral function.

One has only to consider the many means devised by nature, to maintain equability of pressure within the cranium to appreciate the dangers of fluctuation in the cerebral blood streams, for in addition to the admirably adapted automatic apparatus just mentioned, every anatomist has been struck with admiration by the tortuous course of the internal carotid and vertebral arteries, which protect the brain from the shock of the ventricular impulses; the unique arrangement of the cerebral veins and sinuses, which form a quasi overflow; the ebb and flow of the cerebro-spinal fluid, and the intricate and ever alert vaso-neuro-mechanism, all working in harmony to maintain the integrity of the great nerve centre, and which, though admittedly inadequate to maintain under all circumstances that nicety of adjustment which the extreme delicacy of the brain demands for the performance of its highest functions, yet serve to meet the ordinary requirements of the brain in health. When we contemplate these varied and complex safeguards to the cerebral circulation, in ceaseless operation, to protect the brain from injury and enable it to perform its marvelous functions, it becomes apparent that anything which throws this intricate machinery out of gear, must result in circulatory disturbances and consequent aberrations of cerebral function. That functional perturbations of the cerebrum arising from this cause, eventuate in insanity, will probably not be questioned. And that insanity, thus acquired, is inheritable, rests upon as high a degree of probability as does the inheritability of any other form of insanity, and indeed, while perhaps not so clearly demonstrated, is no less certain than the hereditary transmission of syphilis.

It is not the purpose of this paper to discuss "Evolution," but merely to bring under your notice one of the factors of the wonderful process by which has been evolved the varied and ever-varying Flora and Fauna, which to-day excite our wonder and challenge our admiration. This one factor has been discussed only in so far as seemed necessary to the elucidation of the subject under consideration.

It will suffice, for the purposes of this paper, if we have shown that use tends to strengthen and develop certain parts, as the parts concerned in the locomotion of the race horse, and that disuse diminishes or obliterates them, as in the case of the blind crabs in the Mammoth Cave, in which the eye stalks still remain, or that of the sesamoid bones in the Caucasian, already alluded to, and that modifications thus acquired are transmitted to offspring. For if such modifications do occur, and are inheritable, it is justly inferable that modifications produced by other, and perhaps less obvious functional perturbations, are also inheritable.

Now let us take the case of a female who is suffering from those distressing nervous affections consequent upon perverted ovarian function, such as called into play the genius and skill of Battey.

Now, if the modifications arising from such functional excitation are inherited, and that they are, rests upon evidence that leaves no room for doubt, is not the importance and value of Battey's operation to the individual upon whom it is performed overshadowed by its importance to the State? In other words, is not the operation of Battey, while eminently justifiable in what it accomplishes for the individual operated upon, to be measured rather by what it prevents, than by what it cures? It It would seem that every student of State medicine must answer, yes.

What sadder spectacle can the student of sociology contemplate than the multiplication of offspring in whom are being compounded the miseries of an ancestry who were the victims of ovarian disease? Are there not, then, better grounds for the performance of Battey's operation than those for which its illustrious founder instituted it? This is admittedly debatable ground, and through due deference for the opinion of those whose reasons for dissenting are sacred, I do not press the question.

But let us take another illustration, for example, that of a uterus in which there is a cervical laceration and sub-involution, followed by displacement, and giving rise to the distressing train of symptoms familiar to every gynecologist.

Is it any longer a matter of doubt that the modifications flowing from these altered functions are transmitted to offspring? Aside from the theoretical considerations already alluded to, and which, of themselves, would seem to settle the question, clinical observations leave no room for doubting the inheritability of such modifications. Tnis much, then, being taken for granted, who can estimate the benefits to posterity of the operations of Emmet and Alexander, and all other surgical procedures which result in the restoration of normal functions, or an approximation thereto? An interesting and striking example of functional and structurral modifications that may be brought about by surgery, is afforded by some of the major operations; especially is this the case in amputations of the lower extremities. These amputations, when near the trunk, are frequently followed by decided improvement in the health and strength and bodily weight of the person operated upon. A number of cases have come under my personal observation, in which persons who had sustained amputations at or near the hip joint, during the iate war, had become robust and plethoric, who prior to such amputations were frail and anæmic. So many cases of this kind have been observed as to leave no room for doubt that the improvement may be fairly attributed to the amputations.

In these cases the nutritive functions, prior to the amputations, were so impaired as to be insufficient to meet the requirements of the organism in its entirety, but with the lopping off of a large portion of the body, (about one-third of the body weight in amputations at the hip joint,) the area of distribution of nutrient matter would be greatly reduced. Hence, if the nutrient functions remained the same as before the operation, improvement in nutrition must certainly follow; but it is perfectly clear that with the increase ot the blood supply to the various parts, improvement in the functions of these parts would ensue. Indeed, the functional changes induced in the entire organism would be very great. The viscera, which before the amputation received a supply of blood inadequate for the proper performance of their functions, would now be amply supplied, and improved digestion, absorption, assimilation and dissimilation would be the result. It seems perfectly obvious that the physical and psychical modifications occurring in these cases are the outcome of functions which the surgical measures adopted have restored to the normal, or approximately normal, standard, and which prior to the operation were far below normal.

I am not unmindful of the inadequacy, in the present state of surgical science, of the means necessary to the restoration of normal function in many surgical cases in which there is perverted function. But this in no way affects the argument, which is that surgical skill should be applied as well to the prevention as to the cure of surgical diseases, and that it is the province and duty of the surgeon, a duty he owes to posterity, to exhaust surgical science in the endeavor to bring back to the normal performance of function any part or organ which may be functionating abnormally, to the end that the terrible consequences of perverted functions may not be visited upon offspring.

"Necessity is the mother of invention," says an old adage, and to urge the necessity of preventive surgery, a surgery that contemplates extending its benefits to unborn generations, is the main object of this paper. With this necessity felt and properly appreciated, the means will, to a large extent, be developed as surely as that water will find its level, or air a vacuum.

Even now the means at our command, if employed with a view to preventing the transmission of undesirable modifications, would, it may be safely assumed, result in much good to coming generations; and while greatly enlarging the field of legitimate surgery, would lead to the improvement of surgical art and the expansion of surgical science.

The operation for the radical cure of hernia, in respect of freedom from danger and perfection of results, certainly leaves much to be desired; but the brains and hands of men will not rest until this operation is shorn of danger, and its results are such as to stamp out this prevalent and growing scourge.

I shall not venture an opinion as to whether it would be wise in the present state of surgical science to perform the operation for the radical cure of hernia in all cases in which the application of a suitable truss has resulted in failure to cure; but if it is true, as I feel sure it is, that the tendency to hernia is accumulating through heredity, under the temporizing methods now practiced, and that this tendency would be diminished or abolished by the radical operation, when successful, there would be much better and broader grounds for instituting radical measures than where no other incentive to such operation existed than the prospect of benefiting alone the patient operated upon.

But these glimpsing details and brief comments may become wearisome, and I must be content with this hasty and imperfect sketch. I hope it will be sufficient to indicate the grounds which, to my vision, appear large and inviting, a broad and fruitful field, as yet unexplored, for preventive surgery. I trust there is a good day coming, in which lovely woman will be able to lay aside the abominable devices which inherited infirmities now doom her to wear, and in which man may put away forever the torturing truss, which is now necessary to enable him to live and "bring forth seed after his kind."

I trust you will agree that the reasons for the grounds taken in this paper, no matter how insufficient they may appear to you, have been fairly stated, and not with immodesty. The man whose mind has been trained to scientific habits of thought needs no admonition to make him distrustful of the generalizations of others, or to remind him of the fallibility of his own. The conclusions arrived at seem to my mind to be fully in accord with the established facts and logical inferences of natural science, and if I am right in this, it is not too much to say that the claims of preventive surgery are well worthy the attention of the medical profession.

Some years ago Professor Huxley said: "On the evidence of paleontology, the evolution of many existing forms of animal life from their predecessors is no longer an hypothesis, but an historical fact; it is only the nature of the physiological factors to which that evolution is due which is still open to discussion." If this statement of this able, cautious and conscientious observer was justifiable when uttered, what shall we say of it to-day, when the accumulated revelations of paleontology have added their whole weight of testimony in its favor? Certainly it is not too much to say that the men whose special scientific training best fit them to form an opinion upon the subject, unanimously agree with Huxley.

Assuming, from this high authority, that "the evolution of many existing forms of animal life from their predecessors is no longer an hypothesis, but an historical fact," the question arises: Is the physiological factor, upon which we have laid so much stress, viz.: the hereditary acquirement of functionally produced modifications, a true cause in bringing about certain evolutional changes? For upon this factor hinges the argument we have attempted to make. If this be true, the conclusion would seem to be inevitable that changes of functions resulting from pathological or abnormal conditions must also produce inheritable modifications; and the further conclusion would also seem to be justifiable that, where surgery can intervene and restore affected parts or organs to that condition in which they will physiologically perform their functions, the transmission of pathological modifications must sooner or later cease.

